# Supplementary material for: Comparison of Performance in the Six-Minute Walk Test (6MWT) between Overweight/Obese and Normal-Weight Children and Association with Haemodynamic Parameters: A Cross-Sectional Study in Four Primary Schools
Source: Nutrients. 2024 Jan 25;16(3):356. doi: 10.3390/nu16030356 (PMC10857357; doi:10.3390/nu16030356)
Supplement: Supplementary file 1 [file nutrients-16-00356-s001.zip › nutrients-2829858-supplementary.pdf]

Table S1 Relation between the fatty acids and haemodynamic parameters measured after the 6MWT

	Post-6MWT SBP	Post-6MWT Z-SBP	Post-6MWT DBP	Post-6MWT Z-DBP	Post-6MWT HR	Post-6MWT RPP	Post-6MWT $SatO_2$	6MWT distance
C16:1n7 (palmitoleic)	**0.168***	**0.164***	**0.213****	**0.177****	0.101	0.154	-0.149	**-0.150***
C16:0	**0.201****	**0.232****	0.002	0.002	0.044	**0.130***	-0.080	0.001
C18:0	**-0.154***	**-0.163***	-0.010	-0.013	-0.065	0.094	-0.118	0.052
C18:1n9	0.071	0.110	-0.018	0.005	0.011	-0.107	0.043	-0.066
C18:2n6 LA	-0.002	0.004	-0.031	-0.026	0.044	0.063	0.031	-0.062
C18:3n6 GLA	-0.080	-0.066	-0.095	-0.072	-0.042	0.125	-0.073	-0.127
C20:3n6 DGLA	0.072	0.079	-0.061	-0.050	0.082	-0.067	0.096	0.080
C20:4n6 AA	-0.015	-0.052	0.008	-0.015	-0.049	-0.066	-0.048	0.006
C18:3n3 ALA	-0.124	-0.130	-0.096	-0.089	-0.140	0.029	-0.154	0.004
D5D	-0.069	-0.088	0.045	0.032	-0.085	-0.098	0.045	-0.051
D6D	-0.076	-0.062	-0.075	-0.053	-0.036	-0.067	0.120	-0.108
SDC-16	**0.137***	**0.130***	**0.216****	**0.177****	-0.142	0.087	0.139	**-0.154***
SDC-18	0.053	0.089	0.008	0.032	-0.170	-0.003	0.082	-0.048

6MWT, 6-minute walk test; AA: arachidonic acid; ALA: Alpha-Linolenic-Acid; DBP: Diastolic Blood Pressure; DGLA: Dihomo-Gamma-Linolenic-Acid; FA: Fatty Acids; GLA: Gamma-Linolenic-Acid; HR: Heart Rate; LA: linoleic acid RPP Rate Pressure Product, SBP: Systolic Blood Pressure; D5D: delta-5 desaturase; D6D: delta-6 desaturase.

Table S 2 *Relation between food group intake and haemodynamic parameters measured after the 6MWT*

	Post-6MWT SBP	Post-6MWT Z-SBP	Post-6MWT DBP	Post-6MWT Z-DBP	Post-6MWT HR	Post-6MWT RPP	Post-6MWT $SatO_2$	6MWT distance
Cereals	0.008	0.002	-0.079	-0.095	-0.016	-0.009	-0.027	0.003

Fruit	***0.230****	***0.203****	*0.098*	*0.041*	*0.073*	***0.162****	*-0.114*	*0.011*
Nuts	*0.073*	*0.061*	*-0.027*	*-0.056*	*0.031*	*0.057*	*-0.109*	*-0.021*
Dairy products	*-0.015*	*-0.020*	*0.009*	*0.013*	*0.022*	*0.012*	*0.021*	*-0.001*
Meat	*0.058*	*0.047*	*0.051*	*0.043*	*-0.019*	*0.009*	*0.037*	*-0.064*
Fish	*0.026*	*0.006*	*0.016*	*-0.003*	*0.027*	*-0.009*	*-0.045*	*0.069*
Sweets	*0.057*	*0.043*	*0.001*	*-0.016*	*0.014*	*0.030*	*0.064*	*-0.105*
Junk Food	***0.137****	***0.121****	*0.069*	*0.040*	*-0.023*	*0.042*	*0.029*	*-0.061*

6MWT, 6-minute walk test; DBP: Diastolic Blood Pressure; HR: Heart Rate; RPP Rate Pressure Product, SatO$_2$: oxygen saturation; SBP: Systolic Blood Pressure

Table S3 Relation between anthropometric traits and the differences between hemodynamic parameters pre and post 6MWT (Delta)

	Delta SBP	*Delta DBP*	*Delta HR*	*Delta RPP*	*Delta SatO$_2$*
BMI	***0.132****	*0.018*	***0.186****	***0.226****	*-0.136*
z-BMI	***0.117****	*-0.010*	***0.166****	***0.199****	*-0.025*
Waist/height	*0.080*	*-0.038*	*0.094*	*0.121*	*-0.105*
Waist/hip	*-0.026*	*-0.069*	*-0.010*	*-0.015*	*-0.125*
BSA	***0.157****	*0.036*	***0.169****	***0.227****	*-0.053*
FAT, kg	***0.153****	*0.018*	***0.181****	***0.234****	*-0.169*
FFM, kg	*0.111*	*0.021*	***0.135****	***0.172****	*0.033*
FFM/FM	***-0.181****	*-0.014*	*-0.117*	***-0.182****	*0.024*
TBW	***0.119****	*0.024*	*0.114*	***0.163****	*0.038*

6MWT, 6-minute walk test; DBP: Diastolic Blood Pressure; HR: Heart Rate; RPP Rate Pressure Product, SatO$_2$: oxygen saturation; SBP: Systolic Blood Pressure

Table S*4* Relation between the dietary pattern, physical activity and the differences between hemodynamic parameters pre and post 6MWT (Delta)

	Delta SBP	*Delta DBP*	*Delta HR*	*Delta RPP*	*Delta SatO$_2$*
Healthy Pattern	*0.023*	*0.060*	*0.071*	*0.078*	*-0.044*
Unhealthy Pattern	*-0.006*	*-0.095*	*-0.033*	*-0.027*	*-0.039*

Sedentary activity	*0.026*	*-0.025*	*0.132**	*0.106*	*0.072*
Intense physical activity	*0.055*	*-0.042*	*0.006*	*0.027*	*-0.019*

6MWT, 6-minute walk test; DBP: Diastolic Blood Pressure; HR: Heart Rate; RPP Rate Pressure Product, $SatO_2$: oxygen saturation; SBP: Systolic Blood Pressure

Table S5 Relation between the differences between fatty acids and hemodynamic parameters pre and post 6MWT(Delta)

	Delta SBP	*Delta DBP*	*Delta HR*	*Delta RPP*	*Delta $SatO_2$*
Omega 6 FA	*-0.061*	*-0.085*	*-0.012*	*-0.042*	*-0.104*
Omega 3 FA	*0.064*	*0.110*	*0.053*	*0.058*	*0.060*
Omega 9 FA	*-0.007*	*0.014*	*0.014*	*0.016*	*0.184*
Saturated FA	*0.030*	*0.018*	*-0.037*	*-0.015*	*-0.039*
Trans fatty FA	*0.109*	***0.130****	***0.139****	***0.163****	*0.043*
D5	*-0.027*	*-0.014*	*-0.038*	*-0.048*	*-0.169*
D6	*-0.098*	*0.004*	*-0.113*	***-0.135****	*0.167*
Glucose	*0.045*	*0.024*	*-0.026*	*-0.012*	*-0.026*
Triglycerides	*0.131*	*0.111*	*0.040*	*0.103*	*0.184*
Cholesterol	*-0.074*	*-0.075*	*-0.095*	*-0.123*	*-0.095*

6MWT, 6-minute walk test; DBP: Diastolic Blood Pressure; HR: Heart Rate; RPP Rate Pressure Product, $SatO_2$: oxygen saturation; SBP: Systolic Blood Pressure; D5D: delta-5 desaturase; D6D: delta-6 desaturase.

Linear regressions

S 6 Linear Regression between haemodynamic and anthropometric parameters after adjustment for age, sex, and height

**Post-6MWT SBP**			
	Beta	95%CI	p-value
BMI	0.068	0.037,0.100	<0.001

Waist/Height	1,786	0.274,3.299	0.021
FAT	0.049	0.028,0.070	<0.001
FFM/FM	0.026	-0.006,0.059	0.002
Trans Fatty Acids	0.386	0.026,0.746	0.036
**Post-6MWT DBP**			
BMI	0.049	0.014,0.083	0.006
FAT	0.036	0.013,0.059	0.003
**Post-6MWT HR**			
BMI	0.067	0.033,0,101	<0.001
Waist/Height	1.872	0.450,3.293	0.010
FAT	0.048	0.025,0.071	<0.001
**Post-6MWT RPP**			
BMI	0.084	0.051,0.117	<0.001
Waist/Height	2.120	0.730,3.510	0.003
FAT	0.060	0.038,0.083	<0.001
FFM	0.040	0.006, 0.074	0.023
FFM/FM	-0.045	-0.075,-0.015	0.003
Trans Fatty Acids	0.441	0.048,0.833	0.028

6MWT, 6-minute walk test; DBP: Diastolic Blood Pressure; HR: Heart Rate; RPP Rate Pressure Product, $SatO_2$: oxygen saturation; SBP: Systolic Blood Pressure
